# Cardiovascular Event Reporting in Modern Cancer Radiation Therapy Trials

**DOI:** 10.1016/j.adro.2021.100888

**Published:** 2021-12-29

**Authors:** Rahul N. Prasad, Mark McIntyre, Avirup Guha, Rebecca R. Carter, Vedat O. Yildiz, Electra Paskett, Maryam Lustberg, Patrick Ruz, Terence M. Williams, Onaopepo Kola-Kehinde, Eric D. Miller, Daniel Addison

**Affiliations:** a Department of Radiation Oncology, Arthur G. James Cancer Hospital and Richard J. Solove Research Institute, Ohio State University Comprehensive Cancer Center, Columbus, Ohio; b Cardio-Oncology Program, Division of Cardiology, Ohio State University Medical Center, Columbus, Ohio; c Harrington Heart and Vascular Institute, Case Western Reserve University, Cleveland, Ohio; d Center for the Advancement of Team Science, Analytics, and Systems Thinking (CATALYST), Ohio State University College of Medicine, Columbus, Ohio; e Center for Biostatistics, Department of Biomedical Informatics, Ohio State University, Columbus, Ohio; f Division of Cancer Control and Prevention, Department of Internal Medicine, College of Medicine and Comprehensive Cancer Center, Ohio State University, Columbus, Ohio; g Department of Breast Medical Oncology, Yale Cancer Center, New Haven, Connecticut; h Department of Radiation Oncology, City of Hope Comprehensive Cancer Center, Duarte, California

## Abstract

**Purpose:**

Cardiovascular disease (CVD) is the leading cause of morbidity and mortality in cancer survivors, particularly after chest radiation therapy (RT). However, the extent to which CVD events are consistently reported in contemporary prospective trials is unknown.

**Methods and Materials:**

From 10 high-impact RT, oncology, and medicine journals, we identified all latter phase trials from 2000 to 2019 enrolling patients with breast, lung, lymphoma, mesothelioma, or esophageal cancer wherein chest-RT was delivered. The primary outcome was the report of major adverse cardiac events (MACEs), defined as incident myocardial infarction, heart failure, coronary revascularization, arrhythmia, stroke, or CVD death across treatment arms. The secondary outcome was the report of any CVD event. Multivariable regression was used to identify factors associated with CVD reporting. Pooled annualized incidence rates of MACEs across RT trials were compared with contemporary population rates using relative risks (RRs).

**Results:**

The 108 trials that met criteria enrolled 59,070 patients (mean age, 58.0 ± 10.2 years; 46.0% female), with 273,587 person-years of available follow-up. During a median follow-up of 48 months, 468 MACEs were reported (including 96 heart failures, 75 acute coronary syndrome, 1 revascularization, 94 arrhythmias, 28 strokes, and 20 CVD deaths; 307 occurred in the intervention arms vs 144 in the control arms; RR, 1.96; *P* < .001). Altogether, 50.0% of trials did not report MACEs, and 37.0% did not report any CVD. The overall weighted-trial incidence was 376 events per 100,000 person-years compared with 1408 events per 100,000 person-years in similar nontrial patients (RR, 0.27; *P* < .001). There were no RT factors associated with CVD reporting.

**Conclusion:**

In contemporary chest RT–based clinical trials, reported CVD rates were lower than expected population rates.

## Introduction

Cardiovascular disease (CVD) is a leading cause of morbidity and mortality after thoracic radiation therapy (RT) and has emerged as a well-recognized limitation of cancer therapy efficacy.^1^ Although some cardiovascular toxic effects are partially attributable to shared risk factors, these characteristics do not entirely explain the severity and frequency of CVD in this population.^2^ Increasingly, unintended RT-associated CVD has been appreciated as an early therapeutic complication, occurring earlier than previously thought. Multiple reports in non-small cell lung cancer (NSCLC) have demonstrated that a significant proportion of cardiac events occur within approximately 2 years of RT completion.^3^^,^^4^ Studies in patients with Hodgkin lymphoma,^5^, ^6^, ^7^, ^8^ breast cancer,^9^, ^10^, ^11^ and NSCLC^3^^,^^12^ treated with RT have reported increased rates of cardiac events that rise with increasing heart dose.^3^^,^^8^^,^^10^^,^^12^ These detrimental effects are pronounced even in locally advanced NSCLC and esophageal cancer, in which survival is measured in years and not decades.^3^^,^^12^, ^13^, ^14^, ^15^ In practice, changes to radiation delivery, including prescription RT dose, fractionation, or target volumes, are typically based on findings from a single, or at most, several landmark studies. Thus, it is imperative that prospective trials accurately measure and report even nononcologic outcomes and safety data. However, whether CVD is consistently reported in contemporary thoracic RT trials is unknown.

## Methods and Materials

Ten leading, representative, high-impact oncology, internal medicine, and RT journals (*Journal of Clinical Oncology; JAMA; JAMA Oncology; JAMA Internal Medicine; The Lancet; The Lancet Oncology; International Journal of Radiation Oncology • Biology • Physics; New England Journal of Medicine; Annals of Internal Medicine*; and *Journal of the National Cancer Institute*) were queried to identify all latter-phase (2 and 3) landmark thoracic RT trials. The search terms *radiation, phase, study, dose, cancer*, and *Gy* (an abbreviation of Gray, joule/kg) were used to identify potential studies. Results were limited to original research only. An additional search term, *chest*, was added to search the *International Journal of Radiation Oncology • Biology • Physics* because without it, nearly every published article in the radiation oncology–specific journal met search criteria. The search results were then manually reviewed, and only phase 2 or 3 trials from 2000 to 2019 wherein chest RT was delivered for lung cancer, esophageal cancer, lymphoma, mesothelioma, or breast cancer in at least 1 arm, with enrollment of greater than 100 patients and a minimum 1-year follow-up, were included. For consistency and because some trials were associated with multiple published articles, only trials reporting initial primary study outcomes were included.

After validation, all additional potentially relevant RT, cancer, and cardiovascular variables within the trials were collected, including year of publication; cancer type; trial phase; funding source; enrollment per arm; trial duration; enrollment period; length of follow-up; patient age, sex, and race; and study endpoints. Cardiovascular disease events were defined as any myocardial infarction, stroke, heart failure (HF), coronary or peripheral revascularization, acute thromboembolism, myocarditis, cardiac tamponade, atrial fibrillation or note of any other arrhythmia (eg, ventricular tachycardia), valvular heart disease, significant hypertension including hypertensive emergency, CVD death, or any other mention of CVD. Variables specific to RT were collected, including the use of sophisticated radiation planning and delivery techniques (intensity modulated radiation therapy), whether dose escalation or de-escalation was tested, the minimum allowable dose in each study arm, and the reporting of heart dose information. Two reviewers independently reviewed clinical trial data before entry. For those trials with ambiguous or missing data, clinicaltrials.gov was queried, and if uncertainty persisted, corresponding authors were contacted for clarification. Because only published data were collected and no patients were involved, institutional review board approval and informed consent were not required.

The primary endpoint was the reported rate of major adverse cardiovascular events (MACEs), defined as myocardial infarction, stroke, HF, coronary revascularization, arrhythmia, or CVD death, across all study participants.^2^^,^^16^^,^^17^ The secondary endpoint was the reported rate of any CVD event across all trial arms. Person-years of follow-up for each trial were estimated by multiplying the median reported follow-up period in each trial by the number of participants. Descriptive statistics were used to summarize patient characteristics, using mean ± standard deviation (SD) or median for continuous variables and frequency counts with percentages for categorical variables. Univariate and multivariable analyses were conducted using multivariable logistic regression, χ^2^ tests, and Fisher exact tests to evaluate the relationships between specific trial characteristics and CVD reporting. For rigor, relationships were assessed using 2 established modeling approaches, including multivariable stepwise regression, with all measured variables meeting a *P* value significance threshold of 0.2 on univariate analysis. Variables remained in the model as long as the *P* value at each stepwise iteration remained at 0.3.^17^ In addition, a second model was created to study the variables significantly associated with nonreporting of CVD in univariate regression. Specific statements about the modeled variables are presented in Table E1. The Hosmer-Lemeshow test was used to assess the parsimony of all models.

Annualized incidence rates of MACEs across all trials were calculated and compared with incidence rates of CVD in a large, contemporary, middle-aged or older population of persons without overt clinical CVD, derived from the prospective, longitudinal Multi-Ethnic Study of Atherosclerosis (MESA) observational cohort study, who were subsequently monitored for development of CVD.^18^ Risk differences were calculated between the observed and expected proportions of reported CVD events within the trial population and were estimated by dividing the observed incidence of CVD among RT trial participants by the reported incidence among the larger population, both per 100,000 person-years of follow-up. A value of <0.8 was considered a low rate of reporting.^17^

Moreover, to further understand potential differences in reporting rates and types of CVD events, observed measures were adjusted for RT-specific factors including method of delivery (intensity modulated radiation therapy) and reported dose (including cardiac dose). All statistical tests were 2-sided, with significance set at α = .05.

## Results

**Fig. 1:**
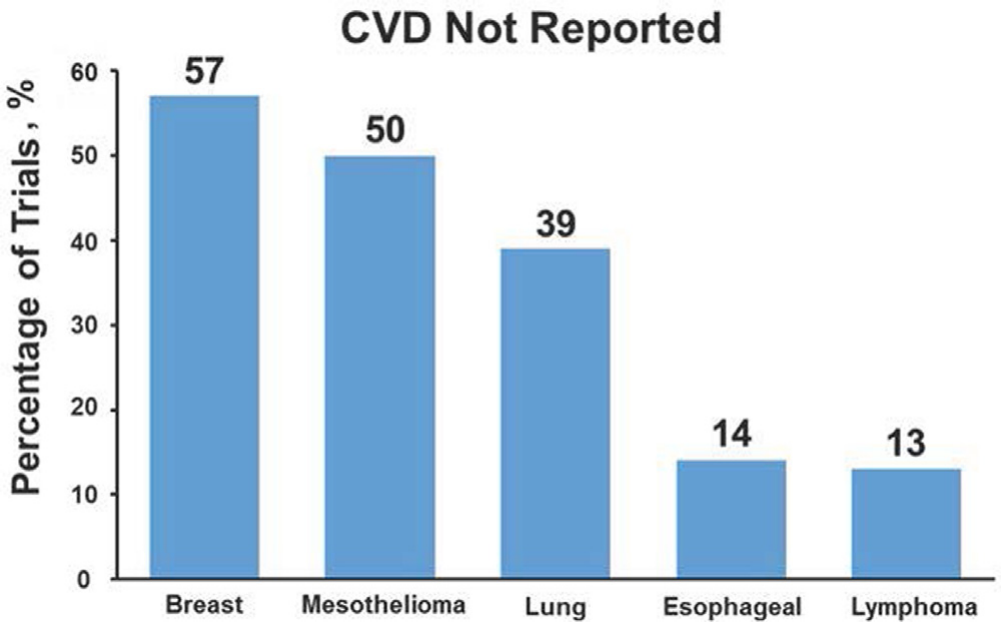
Cardiovascular event nonreporting rate, stratified by cancer type. *Abbreviation:* CVD = cardiovascular disease.

In total, 108 trials were identified, accruing 59,070 patients with 273,587 person-years of follow-up (mean age, 58.0 ± 10.2 years; 46.0% female; Fig. E1). The majority of trials included concurrent immune, biologic, or hormone-based therapy (98.1%), and the median trial size was 100 to 499 participants, with breast and lung cancer accounting for most included trials (Table 1). The Common Terminology Criteria for Adverse Events (CTCAE) was the most frequently used threshold for CVD identification and reporting. Fourteen trials (13.0%) excluded patients with any baseline CVD. Overall, 50.0% of trials did not report MACEs during follow-up, including 37.0% that did not report any CVD events. Nonreporting rates, stratified by cancer type, are depicted in Fig. 1. For each respective form of MACE, more than 75% of trials did not report an occurrence, and the rate of nonreporting did not vary significantly between trials including versus excluding baseline CVD (Table 2). In multivariable analysis, no specific variable was associated with CVD reporting. There was no association between year of trial initiation or mode of RT delivery and CVD reporting.

**Table 1:** Reporting of CVD in follow-up, by cancer trial characteristic

Characteristic	Trials, %	Patients, n	CVD reported, n (%)	*P* value (UVA)	*P* value (MVA)
Overall	108	59,070	68 (63.0)		
Cancer				.01	.056*
Lung	46 (42.6)	17,684	28 (60.9)		
Esophageal	14 (13.0)	3588	12 (85.7)		
Lymphoma	16 (14.8)	9316	14 (87.5)		
Mesothelioma	2 (1.9)	526	1 (50.0)		
Breast	30 (27.8)	27,956	13 (43.3)		
Trial size, participants, n				.38	
100-499	69 (63.9)	17,434	41 (59.4)		
500-999	24 (22.2)	15,763	18 (75.0)		
>1000	15 (13.9)	25,873	9 (60.0)		
Trial phase				.12	
2	26 (24.1)	8155	13 (50.0)		
3	82 (75.9)	50,915	55 (67.1)		
Funding source† (n = 53)				.47	
Industry	20 (37.7)	11,470	13 (65.0)		
Government/nonprofit	20 (37.7)	12,170	15 (75.0)		
Both	13 (24.5)	12,908	9 (69.2)		
Start of enrollment† (n = 107)				.16	
Before 2000	53 (49.5)	24,926	39 (73.6)		
2000-2004	24 (22.5)	16,869	13 (54.2)		
2005-2009	27 (25.2)	15,656	15 (55.6)		
2010-2014	3 (2.8)	1374	1 (33.3)		
Year of publication				.23	
2000-2004	27 (25.0)	13,067	21 (77.8)		
2005-2009	26 (24.1)	10,711	17 (65.4)		
2010-2014	29 (26.9)	19,112	16 (55.2)		
2015-2019	26 (24.0)	16,180	14 (53.8)		
Trial duration, mo				.08	
<36	13 (12.0)	2793	4 (30.8)		
37-48	15 (13.9)	6839	10 (66.7)		
48-60	22 (20.4)	9699	14 (63.6)		
>60	58 (53.7)	39,739	40 (69.0)		
Trial design† (n = 87)				.11	
Superiority	70 (19.5)	31,624	51 (72.9)		
Noninferiority	17 (80.5)	20,511	9 (52.9)		
IMRT allowed† (n = 96)				.72‡	
Yes	9 (9.4)	4138	5 (55.6)		
No	87 (90.6)	42,127	57 (65.5)		
RT dose escalation or de-escalation				.04	.24§
Yes	26 (24.1)	14,396	12 (46.2)		
No	82 (75.9)	44,674	56 (68.3)		
Reported heart dose				.14‡	
Yes	4 (3.7)	1112	1 (25.0)		
No	104 (96.3)	57,958	67 (64.4)		
Met endpoint† (n = 87)				.4	
Yes	28 (32.2)	22,471	21 (75.0)		
No	59 (67.8)	29,664	39 (66.1)		
Median follow-up	48 (12- 135)			.96║	

*Abbreviations:* CVD = cardiovascular disease; IMRT = intensity modulated radiation therapy; MVA = multivariable analysis; RT = radiation therapy; UVA = univariate analysis.

⁎ Multivariable stepwise regression.

† The number was <108 owing to missing data points.

‡ Fisher exact test.

§ Multivariable modeling with variables that achieved significance.

║ Kruskal-Wallis *P* value.

**Table 2:** Rate of nonreporting by key types of CVD, stratified by trials excluding CVD and trials not reporting CVD

Type of CVD	Rate of nonreporting among all trials, n (%)	Rate of nonreporting among trials reporting CVD, n (%) (N = 68)	Rate of nonreporting among trials including patients with CVD, n (%) (N = 94)	Rate of nonreporting among trials excluding patients with CVD, n (%) (N = 14)	*P* value*
Heart failure	84 (77.8)	44 (64.7)	72 (76.6)	12 (85.7)	.73
Acute coronary syndrome	88 (81.5)	48 (70.6)	78 (83.0)	10 (71.4)	.28
Arrhythmia	96 (88.9)	56 (82.4)	84 (89.4)	12 (85.7)	.65
Cerebrovascular accident	89 (82.4)	49 (72.1)	78 (83.0)	11 (79.6)	.71
Cardiac arrest or SCD	98 (90.7)	58 (85.3)	86 (91.5)	12 (85.7)	.62

*Abbreviations:* CVD = cardiovascular disease; SCD = sudden cardiac death.

⁎ Fisher exact test.

**Fig. 2:**
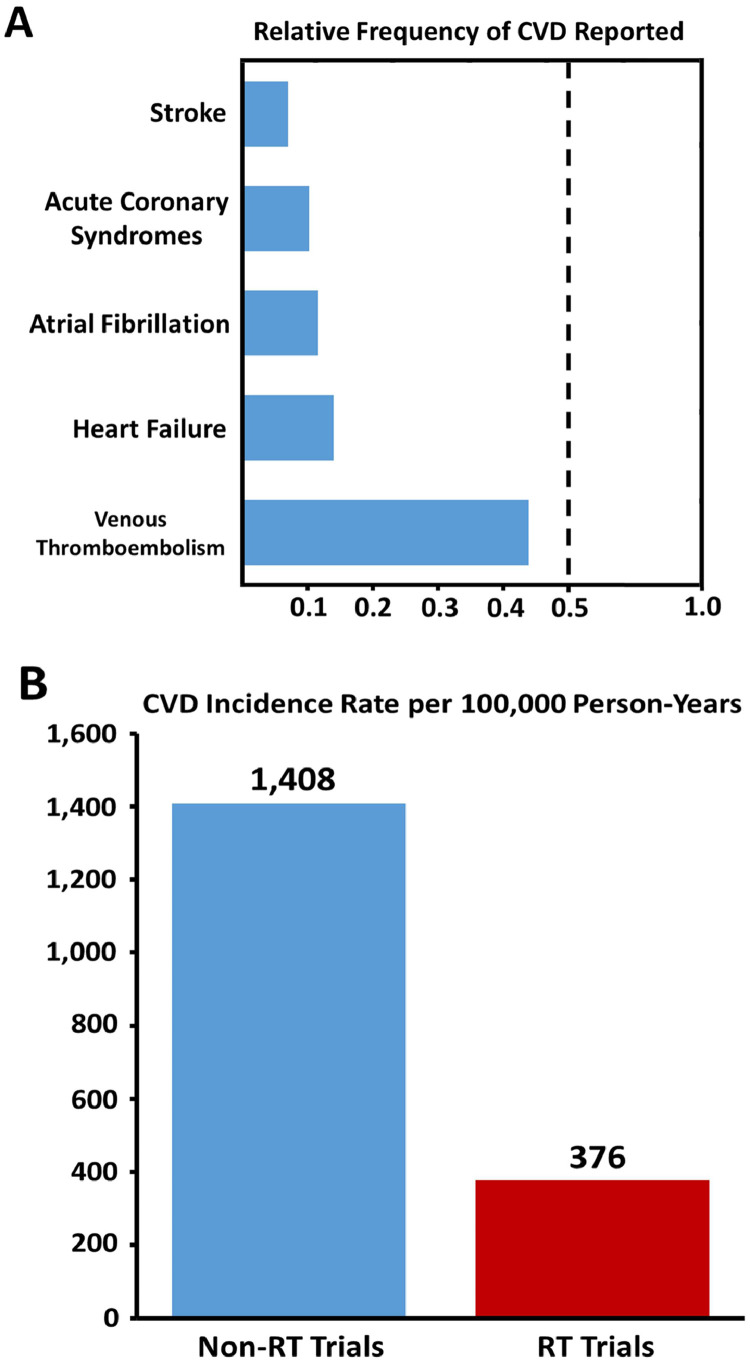
**(A)** Relative frequency of CVD events reported in cancer trials with respect to a representative cohort derived from the Multi-Ethnic Study of Atherosclerosis. **(B)** Comparison of major adverse cardiac event incidence rates between a noncancer population and radiation therapy trial patients. *Abbreviations:* CVD = cardiovascular disease; RT = radiation therapy.

Over the available 273,587 person-years of follow-up (median trial duration, 48 months; range, 12-136 months), 468 MACEs were reported, including 96 HFs, 94 arrhythmias, 75 acute coronary syndrome (ACS) events, 28 strokes, and 20 cardiac-arrest or sudden deaths. The remainder were unspecified grade 3 or higher cardiac toxic effects. Figure 2A shows the relative frequency of CVD events reported in the reviewed trials, compared with rates observed in a similar-aged noncancer trial population free of baseline CVD with a median follow-up of 11 years.^18^ In trials reporting MACEs, the overall relative risk (RR) of HF, ACS, thromboembolic disease, arrhythmia, and stroke was 0.15 (95% confidence interval [CI], 0.13-0.17; *P* < .001), a 6.8-fold lower rate of reported events or a risk difference of 1621 per 100,000 events.^18^, ^19^, ^20^, ^21^, ^22^, ^23^, ^24^

In trials with observed MACEs, 307 were noted in the intervention arms and 144 in the control arms, respectively (RR, 1.96; 95% CI, 1.60-2.39; *P* < .001). The remaining MACE events were not attributable to a specific study arm. Of the 94 trials (87%) that allowed patients with baseline CVD, 47 (50%) did not report any MACEs. Of all categories of examined CVD events, thromboembolic disease was the most commonly reported event type. Furthermore, of the 40 trials that did not report any CVD at initial publication, 5 (4.6% of all trials) reported CVD in follow-up secondary analyses.

**Fig. 3:**
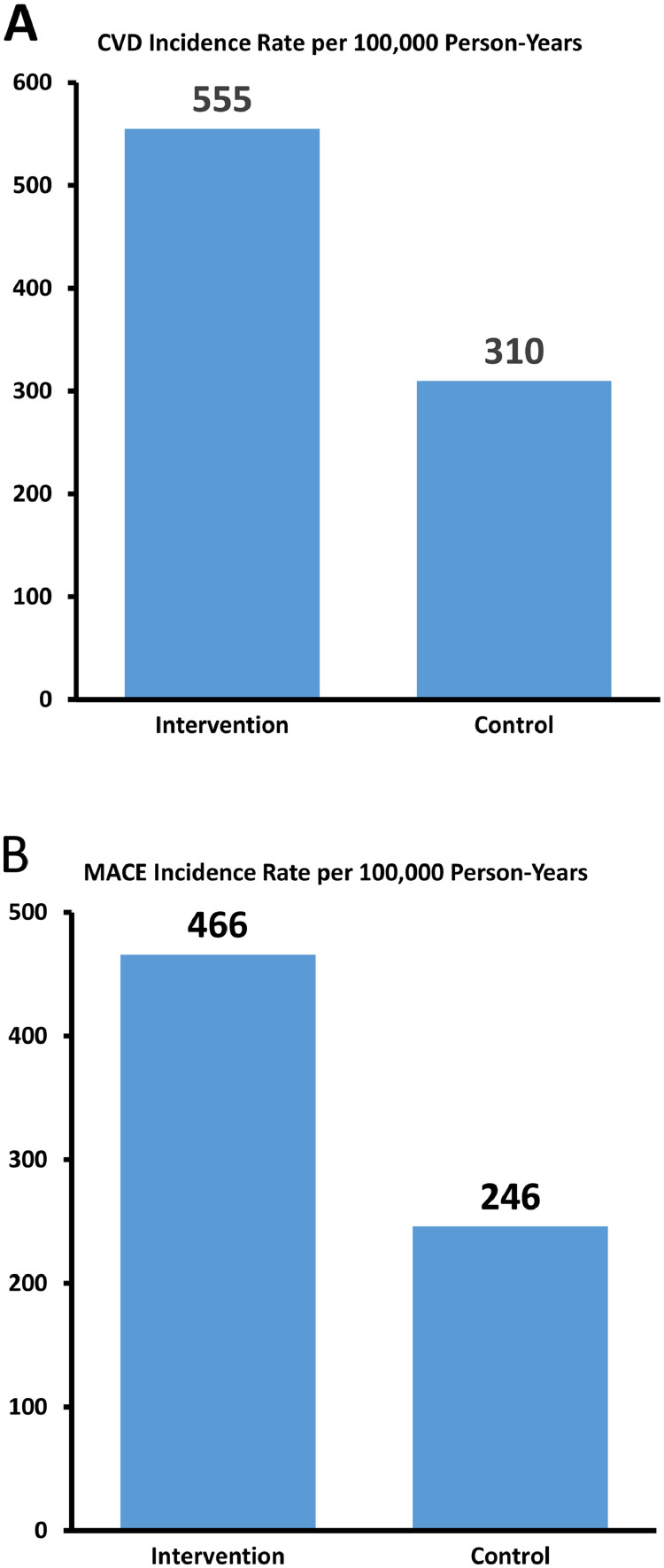
**(A)** Comparison of the reported rate of all cardiovascular events, by trial arm, across all trials. **(B)** Comparison of the reported rate of MACEs, by trial arm, across studies reporting MACEs. *Abbreviations:* CVD = cardiovascular disease; MACE = major adverse cardiac event.

Among all 108 trials, regardless of inclusion or exclusion of baseline CVD, 1221 CVD events (MACE and non-MACE) were noted, with 791 in the intervention arms and 407 in the control arms (RR, 1.78; 95% CI, 1.58-2.01; *P* <.001; intervention-to-control). This corresponds to a total CVD rate of 555 versus 310 per 100,000 person-years in the intervention and control arms, respectively (Fig. 3A). Furthermore, the 468 MACEs (out of 1221 total CVD events) during a cumulative 124,432 years of trial patient follow-up from the 50.0% of trials reporting MACEs correspond to an overall weighted average reported MACE incidence rate of 376 per 100,000 person-years (466 vs 246 per 100,000 person-years in the intervention and control arms, respectively; Fig. 3B). This rate yielded a RR of 0.27 (95% CI, 0.24-0.30; *P* < .001), translating into a 3.75-fold lower rate of reported events and a risk difference of 1032 per 100,000 person-years compared with the reported incidence rate of 1408 per 100,000 person-years in a similar comparison population (Fig. 2B).^18^

In the 14 trials enrolling participants without any baseline CVD, 51 MACE events were reported, including 22 HFs, 9 arrhythmias, 7 ACS events, 2 strokes, and 2 cardiac-arrest or sudden deaths. In this subset, the overall RR of HF, ACS, thromboembolic disease, arrhythmia, and stroke was 0.065 (a 15-fold lower rate of reported events; *P* < .001) compared with rates in a similar-aged noncancer trial population free of baseline CVD.^18^ Altogether, 50.0% of these trials did not report any MACE events, and 10.2% did not report any CVD events. In total, 133 CVD events (MACE and non-MACE) were noted, with 72 in the intervention arms and 56 in the control arms. The 51 MACE events during a total of 29,911 years of patient follow-up translate to an observed rate of MACEs of 171 per 100,000 person-years (218 per 100,000 person-years in the intervention arms).

## Discussion

In this evaluation of contemporary landmark trials involving chest RT, nearly 40% of trials did not report any CVD events during follow-up. This pattern remained even after accounting for RT-delivery strategies and underlying cancer disease type. Similarly, in multivariable analysis, no variables predicted nonreporting of CVD, providing further evidence that underreporting is a systematic issue not easily attributable to any single factor or handful of factors. Rates of MACEs were substantially lower than observed rates in a comparable general population without preexisting CVD, at just over one-fourth of expected population rates.^18^ Low reported rates of CVD events persisted even after including or excluding patients with baseline CVD, suggesting systematic underreporting or underappreciation of CVD events among participants in oncology trials involving chest RT. This is concerning, because most cancer patients demonstrate elevated rates of cardiac risk compared with broader populations.^16^ Because survival rates increase with improving therapy,^1^ recognizing CVD becomes increasingly important, particularly with novel pharmaceuticals conferring additional cardiovascular risk.^25^

There is growing evidence suggesting RT-associated cardiotoxicity is an earlier issue even in cancer patients with a previously poor prognosis, including those with NSCLC.^3^ Despite the significant risk of short-term cancer-related mortality in locally advanced NSCLC, a large retrospective single-institution study noted a 1-year MACE rate of 2.5% in patients treated with thoracic RT, rising to 8% to 10% by 5 years.^3^ Notably, even with dramatically increasing NSCLC survival, within the current analysis, nearly 40% of lung trials did not report any CVD. Driven by toxicity concerns, considerable efforts to de-escalate treatment of common chest malignancies through omission of RT have occurred.^26^^,^^27^ However, we found that CVD was inconsistently reported in RT-based trials. For example, studies in Hodgkin lymphoma survivors treated with mediastinal irradiation before 2000 show high cardiac risk even 5 years posttreatment, with a subsequent 16% cumulative incidence rate.^6^ Risks are highest in patients also receiving anthracycline-based systemic therapy, demonstrating that multimodal therapy, although frequently necessary, has the potential for additional toxic effects.^7^ The lymphoma studies in our analysis had the lowest CVD nonreporting rate of any cancer, but even among these studies, nearly 1 in 8 did not report CVD.

These findings are supported even by non-RT–based analyses, wherein inconsistent CVD reporting in anticancer trials was observed.^28^ However, our analysis found even larger gaps in reported events in breast cancer trials, incongruous with historical data suggesting a small but significantly increased risk of CVD in patients with breast cancer receiving RT.^9^, ^10^, ^11^ Increased absolute cardiac mortality rates of 0.3% in nonsmokers and 1% in smokers after RT have been seen,^11^ with increasing cardiac dose linked to rising event rates, including a linear increase in major coronary events of 7.4% per Gy.^10^ Like in lymphoma, these historical studies exposed patients to heart doses unacceptable by modern standards, but more recent data documented an increased rate of acute coronary events of 16.5% per Gy even with a mean heart dose of only 2.37 Gy.^29^ Thus, it is notable that 56.7% of breast trials with extended follow-up in this study did not report cardiac events. Absolute baseline and post-RT risks for MACEs vary by population. However, growing population-level data and available large case-control studies^9^, ^10^, ^11^ suggest that cardiac events occur with enough frequency that at least some major (or minor) events may be noted in those individual trials with large sample sizes and/or multiyear follow-up.

Physicians, patients, and families use trial data to weigh the risks and benefits of therapy carefully; these stakeholders rely on consistent reporting.^17^ Reporting of toxic effects in cancer clinical trials uses standardized toxicity grading scales; however, this study found significant variability in the reporting rate of CVD events, consistent with known underreporting in pharmaceutical trials.^17^^,^^28^ Proposed contributing factors in pharmaceutical trials include narrow trial scope,^17^ researcher bias,^28^ and misinterpretation of signs and symptoms,^28^ and these factors are likely relevant to RT trials. Most included trials had oncologic primary endpoints. Even if toxic effects were addressed in secondary endpoints, if these studies too narrowly focused on efficacy outcomes at the expense of careful monitoring for toxic effects, CVD rates might be systematically underrepresented.^17^^,^^28^ Furthermore, research biases may play a role in underreporting, and publication bias favoring positive trials offering favorable risk-to-reward ratios for investigative therapies should be considered.^28^ Additionally, specialist clinicians may not as consistently identify and interpret the subjective manifestations of CVD required to adequately catalog toxic events as would practitioners specialized in these conditions,^17^ or nonspecific symptoms may be incorrectly attributed to other disorders.^28^ Studies have also shown that clinicians detect fewer adverse events associated with anticancer drugs than do patients,^28^ and this may hold true with RT effects. Although overall survival outcomes are objective, CVD mortality may be incorrectly attributed to other causes owing to the inherent complexity of assigning cause of death in patients with comorbidities.^17^ The authors of the earliest included trials, which began accruing patients in the 1990s, may have been less aware of cardiac toxic effects, owing to worse overall survival in cancer populations at that time or less available risk data. However, we found no significant association between CVD reporting and the era of trial initiation. Potential interventions to address these issues include emphasizing patient-centered toxic outcomes, use of centralized event adjudication committees, posttrial analysis of the patient-specific *International Classification of Diseases* code event entries, and expanded CVD screening.^17^^,^^28^

### Limitations

Several limitations of this study should be acknowledged. Cancer populations were heterogeneous, which may have affected true rates of cardiac events. For consistency, we focused on initial trial reports, and a few trials may have reported additional toxicity data in subsequent iterations, possibly magnifying the degree of observed underreporting relative to a comparison population. However, we reviewed secondary analyses from these trials and found that similar patterns of nonreporting remained, suggesting that this decision likely had a minimal effect on our findings. Additionally, evaluating only initial publications does not explain the elevated rate of entirely nonreported CVD events, because nearly all studies incorporated extended follow-up, wherein occurrence of at least 1 CVD event would be likely. Furthermore, inclusion of subsequent reports would have introduced further heterogeneity to this analysis, because not all studies yield multiple publications, and in lung cancer, the most detailed data on toxic effects may be presented at the time of initial publication owing to poor patient prognosis precluding extended follow-up.

Additionally, we focused on phase 2 or later trials with definitive intent and minimum requirements for enrollment and follow-up duration. Data from palliative, phase 1, smaller, or shorter-term studies were excluded, because we wished to investigate cardiac reporting consistency in the trials most likely to influence clinical practice standards. However, it is unlikely that trials with smaller patient populations or shorter follow-up would report rates of CVD that are more consistent with those in the general population than would more extensive, rigorous studies. Inherent to the trials themselves is the limitation that rather than using objective metrics, CVD was defined by administering standardized but subjectively estimated CTCAE grades, as commonly used by investigators. Moreover, stringent enrollment criteria lead to clinical trial participants often being healthier than real-world counterparts.^28^ However, this study showed that low CVD reporting persisted whether or not trials included patients with prior CVD. Limiting the validity of our comparison cohort is the lack of a valid, oncology-specific comparison population.^25^ Given that only 14 included trials excluded baseline CVD, a population of general middle-aged patients free of baseline CVD is an imperfect control, because it likely underestimates expected event rates among patients with cancer.^30^ However, we found that the rate of MACEs in the subset of trials excluding baseline CVD was consistent with the rate of MACEs across all 108 included trials. Additionally, the MESA cohort does not perfectly match the cardiovascular risk distribution of our pooled trial population. However, the sex (46% female in RT trials and 50% in the MESA study) and age (mean [SD], 58 [10] years in RT trials and 45-84 years in the MESA study) distributions are comparable, and more exact comparison cohorts were not readily available. Furthermore, it is unknown whether prophylaxis through medications such as aspirin or statins or lifestyle modifications including exercise or dietary choices may improve post-RT outcomes. As such, prospective cardiac-focused intervention studies after RT are needed.

## Conclusion

In summary, patients treated with RT face the potential for unintended CVD. Cardiotoxicity remains an obstacle to optimizing long-term cancer outcomes. In contemporary chest RT–based trials, cardiac event rates appear to trail expected rates in the general population. Further research into the systematic nature of CVD awareness and reporting in oncologic RT-based trials is needed.

## Appendix Supplementary materials

Image, application 1

## Footnotes

Supplementary material associated with this article can be found in the online version at doi:10.1016/j.adro.2021.100888.
