# Supplementary material for: Cardiovascular Event Reporting in Modern Cancer Radiation Therapy Trials
Source: Adv Radiat Oncol. 2021 Dec 29;7(2):100888. doi: 10.1016/j.adro.2021.100888 (PMC8844682; doi:10.1016/j.adro.2021.100888)
Supplement: Supplementary file 1 [file mmc1.pdf]

Supplementary Figure 1. CONSORT diagram. *additional search term chest was used to query IJROBP. Gy = Gray. JCO = Journal of Clinical Oncology. JAMA = Journal of the American Medical Association. IJROBP = International Journal of Radiation Oncology, Biology, Physics. NEJM = New England Journal of Medicine. IM = Internal Medicine. JNCI = Journal of the National Cancer Institute

Query of the online search function of 10 high impact journals' archives from 2000-2019 using terms* "radiation," "phase", "study", "dose", "cancer," and "Gy" (n = 2,468)

JCO	JAMA	JAMA Oncology	JAMA IM	Lancet	Lancet Oncology	IJROBP	NEJM	Annals of IM	JNCI
N = 869	N = 16	N = 35	N = 1	N = 43	N = 126	N = 1,256	N = 35	N = 1	N = 86

Inclusion only of first reports from definitive phase II/III trials where radiation was delivered in at least 1 arm for lung, esophageal, lymphoma, mesothelioma, or breast cancer with at least 1-year median follow up and 100 enrolled patients (n = 108)

JCO	JAMA	JAMA Oncology	JAMA IM	Lancet	Lancet Oncology	IJROBP	NEJM	Annals of IM	JNCI
N = 54	N = 0	N = 2	N = 0	N = 10	N = 15	N = 17	N = 5	N = 0	N = 5

Supplementary Table 1. Model Statements for Multivariable Analysis

Model	Included Variables	Significant Variables	OR	CI -ul	CI-ll	p-value
Model 1	Cancer, Trial Size, Trial Phase, Funding Source, Start of Enrollment, Year of Publication, Trial Duration, Trial Design, IMRT Allowed, Dose Escalation/De-escalation, Heart Dose reported, Met Endpoint	Trial Design	0.225	0.058	0.865	0.035
	Cancer, Trial Phase, Start of Enrollment, Trial Duration, Dose Escalation	None				0.133
	Cancer, Trial Phase, Start of Enrollment, Trial Duration	None				0.104
	Cancer, Trial Phase, Start of Enrollment	None				0.104
	Cancer, Trial Phase	None				0.056
Model 2	Dose Escalation, Cancer, Trial Phase	None				0.24

CI = confidence interval

IMRT = intensity modulated radiation therapy

OR = odds ratio
